# Associations of Bisexuality and Homosexuality with Handedness and Footedness: A Latent Variable Analysis Approach

**DOI:** 10.1007/s10508-018-1346-9

**Published:** 2018-11-29

**Authors:** Ulrich S. Tran, Michael Kossmeier, Martin Voracek

**Affiliations:** Department of Basic Psychological Research and Research Methods, School of Psychology, University of Vienna, 1010 Vienna, Austria

**Keywords:** Sexual orientation, Handedness, Footedness, Prenatal testosterone, Geschwind–Galaburda theory, Callosal hypothesis

## Abstract

Non-right-handedness appears to be more common among bisexuals and homosexuals than among heterosexuals, which might be indirect evidence of effects of prenatal androgen exposure. Current data suggest higher prenatal testosterone levels among bisexual and homosexual women, but are inconclusive for men. This study examined the association between sexual orientation and non-right-handedness for sex differences and whether higher rates of mixed-handedness, rather than left-handedness, might be the driving factor. This allowed for more specific tests regarding the predictions of two competing theories of prenatal androgen exposure, the Geschwind–Galaburda theory and the callosal hypothesis, than in previous research. Being a potentially better indicator of cerebral lateralization than handedness, associations with footedness were also explored. To counter inconsistencies and shortcomings of previous research, we utilized two large discovery and replication datasets (*n*s = 2368 and 1565) and applied latent variable analysis to reliably classify lateral preferences (i.e., handedness, footedness). This maximized the statistical conclusion validity and allowed for direct tests of replicability. Sexual orientation was differentially associated with lateral preferences among men and women. Associations among women were consistent with predictions of the Geschwind–Galaburda theory, whereas among men they were consistent with predictions of the callosal hypothesis. The results were further consistent with models of homosexuality that suggest a role of parental epigenetic marks on sexually dimorphic fetal development. Research efforts should be increased with regard to footedness and epigenetic theories of homosexuality.

## Introduction

Lateral preferences refer to the preference for using the limbs or organs located on one side of the body, such as hands, feet, eyes, or ears, over the limbs or organs on the other side of the body. Handedness—the preference of one hand over the other for various tasks—is one easily observed lateral preference that has long been scientifically studied, and assumed to be an indicator of cerebral lateralization (i.e., the tendency of brain or cognitive functions to be more dominant in one brain hemisphere than in the other). Handedness is also linked with sexual orientation; i.e., bisexual and homosexual individuals report higher rates of non-right-handedness than heterosexual individuals (e.g., Blanchard & Lippa, 2007; for meta-analytical evidence, see Lalumière, Blanchard, & Zucker, 2000).

There is evidence that homosexuality has genetic underpinnings (e.g., Sanders et al., 2015), but a wealth of data also suggests associations between sexual orientation and prenatal androgen action (for an overview, see Balthazart, 2012). Neurohormonal theory assumes that for men lower prenatal testosterone levels and for women higher prenatal testosterone levels during critical developmental stages are associated with later bisexuality/homosexuality, as they entail a relative feminization in the male fetus and masculinization in the female fetus. Alternatively, lower testosterone sensitivity could be the responsible mechanism among men (Balthazart & Court, 2017; Breedlove, 2017).

Prenatal androgen exposure might also explain the association between sexual orientation and handedness. The Geschwind–Galaburda theory (GGT; e.g., Geschwind & Galaburda, 1987) posits that high prenatal testosterone levels cause a delay in the fetal development of the left cerebral hemisphere which results in a right-hemisphere dominance and hence in a tendency for left-handedness. According to the GGT, high prenatal testosterone levels entail not only a masculinization of the female fetus, but also a feminization of the male fetus (contrary to neurohormonal theory). Overall, the male fetus is subjected to higher levels of intrauterine testosterone than the female fetus. The GGT is thus consistent with the higher prevalence of left-handedness among men than among women (Papadatou-Pastou, Martin, Munafò, & Jones, 2008). A recent large-scale study also confirmed a further prediction of the GGT, concerning an unequal birth month distribution of left-handed men (Tran, Stieger, & Voracek, 2014a).

The callosal hypothesis (CH; Witelson & Nowakowski, 1991) applies to men only and assumes, in line with neurohormonal theory, that low prenatal testosterone levels are associated with later homosexuality. According to the CH, high prenatal testosterone enhances processes of cerebral lateralization through mechanisms of axonal pruning, thereby resulting in stronger left-hemisphere dominance and a smaller corpus callosum. Lower testosterone levels entail a larger corpus callosum which is assumed to be linked with feminization, and thus male homosexuality, in the CH. Consistent with this, women have a larger corpus callosum than men (Shiino et al., 2017), and there is evidence that right-handed homosexual men have a larger corpus callosum than right-handed heterosexual men (Witelson et al., 2008). Higher cord-blood testosterone concentrations (Hollier, Maybery, Keelan, Hickey, & Whitehouse, 2014) are further linked with stronger left-hemisphere lateralization of language among men. Regarding associations with handedness, the CH tentatively assumes a link between a larger corpus callosum and non-right-handedness. As the CH makes no predictions for women, its relationship with the higher prevalence of left-handedness among men than among women remains undefined. Neurological studies suggest that callosal size might be associated with a lower strength (degree) of hand preference, but not with its direction (right versus non-right; Luders et al., 2010); i.e., a larger corpus callosum might be associated with mixed-handedness (the relative lack of a clear hand preference), but not with left-handedness. Yet, the CH also leaves open the possibility that there might be no association between callosal size and handedness in homosexual men at all (Witelson et al., 2008).

Studies that investigated both male and female samples mostly reported a weaker, and sometimes even no, association between sexual orientation and non-right-handedness among men (Blanchard & Lippa, 2007 [using the numbers presented in Table 1]; Ellis, Skorska, & Bogaert, 2017; Lalumière et al., 2000; Xu & Zheng, 2017; Yule, Brotto, & Gorzalka, 2014). Only one study reported an association that was stronger among men than among women (Lippa, 2003). The GGT is at odds with this finding, but it might support the CH if there indeed is no association between callosal size and handedness among homosexual men. Further, the driving factor of the association between sexual orientation and non-right-handedness appears to be higher rates of mixed-handedness, but not left-handedness, among bisexual/homosexual individuals (Blanchard & Lippa, 2007; Ellis et al., 2017; Xu & Zheng, 2017). This finding currently needs more evidence. If corroborated, it could provide support for the GGT: Relative right-hemisphere dominance could entail higher rates of both left-handedness and mixed-handedness. Dependent on whether there is an association between callosal size and handedness among homosexual men, it could either support or disprove the CH.

Further indirect evidence from second-to-fourth digit ratio (2D:4D) research suggests that homosexuality is associated with higher prenatal testosterone levels among women, whereas it is associated with either higher or lower levels, probably dependent on ethnicity, among men (Grimbos, Dawood, Burriss, Zucker, & Puts, 2010). Available data concerning the association between salivary, serum or amniotic fluid testosterone levels and handedness do fully support neither the CH nor the GGT (for a review, see Papadatou-Pastou, Martin, & Mohr, 2017).

Thus, the available evidence suggests, albeit not conclusively, that prenatal testosterone levels may be one cause for the association of sexual orientation with handedness. In turn, studies on the association of sexual orientation with handedness could provide indirect evidence for, or against, the effects of prenatal testosterone levels on sexual orientation and could thus help in deciding whether high, or rather low, levels of prenatal testosterone (or, alternatively, lower testosterone sensitivity) might be of relevance in men. However, research on the associations between sexual orientation and handedness appears to be compromised by various methodological and interpretational problems which need to be overcome to arrive at a clearer picture.

### Shortcomings of Previous Research

Many studies employed single-item (e.g., Blanchard & Lippa, 2007; Ellis et al., 2017; Xu & Zheng, 2017) or ad hoc measures of handedness (e.g., Lippa, 2003) or did not distinguish left- from mixed-handers among non-right-handed individuals (e.g., Lippa, 2003; Yule et al., 2014). This may attenuate the reliability of assessment and the validity of results.

Taxometric studies and latent variable analyses provided converging evidence that psychometrically assessed handedness (using validated multi-item inventories) comprises right, left, and mixed preferences (e.g., Dragovic & Hammond, 2007; Tran, Stieger, & Voracek, 2014b). These three preference classes therefore should be differentiated in assessment and analysis as well.

Right-handedness is the dominant norm in the population (around 90% of individuals are right-handed). Hence, large samples are needed. Even so, statistical power still might be insufficient for the separate analysis of men and women. For example, Yule et al. (2014) investigated 969 women, but only 314 men. Further, some studies deliberately oversampled non-heterosexual participants (e.g., Bogaert, 2007; Lippa, 2003; Xu & Zheng, 2017; Yule et al., 2014). While this does increase analytic power, it may also introduce selection bias that may lead to overestimation or underestimation of statistical parameters of interest (see King & Zeng, 2001).

Even though handedness is the most frequently studied indicator of cerebral lateralization, it likely is not the best indicator among the various lateral preferences. Footedness—the preference of using one foot over the other for unipedal or bipedal tasks—is less affected by social stigma and suppression than (left-)handedness is. There is behavioral and psychometric evidence that footedness indeed is a better indicator of cerebral lateralization than handedness (e.g., Elias & Bryden, 1998; Tran et al., 2014b; for an overview of relevant studies, see also Sacco, Di Michele, Semprini, Merni, & Soffitti, 2018). In relation to this, footedness is a stronger marker than handedness for a range of neurodevelopmental disorders, for which links with handedness have previously been established (ADHD: Tran & Voracek, 2018; schizotypy: Tran, Stieger, & Voracek, 2015).

Developmental instability—the increased vulnerability to environmental and genetic stressors during development—is considered a common cause for the associations between neurodevelopmental disorders and handedness. It has previously been linked with sexual orientation as well (Lalumière et al., 2000). Whereas developmental instability might be overall less prevalent among homosexuals than among heterosexuals (for a review of studies, see Kishida & Rahman, 2015), some studies interpreted higher rates of extreme right-handedness among homosexual men as evidence for developmental instability (Bogaert, 2007; Kishida & Rahman, 2015). With regard to this, the associations of lateral preferences with neurodevelopmental disorders, and hence with developmental instability, appear informative: Mixed-handedness, rather than left-handedness or extreme right-handedness, and mixed-footedness have repeatedly been reported as markers of developmental instability in these lines of research (Glover, O’Connor, Heron, Golding, & The ALSPAC Study Team, 2004; Rodriguez & Waldenström, 2008; Tran et al., 2015; Tran & Voracek, 2018). For these reasons, one would thus expect elevated rates of mixed lateral preferences among bisexual/homosexual individuals if developmental instability is truly a causal factor (see also Lippa, 2003).

Even though developmental instability must be considered as an alternative explanation for higher rates of mixed lateral preferences, mixed- and left-footedness were also found to be indicative of testosterone-like effects on the motor abilities in a sport-related context (Tran & Voracek, 2016). Hence, there are multiple and contradictive strands of evidence which need to be carefully weighed in the interpretation of associations between sexual orientation and lateral preferences. Everything else held equal, developmental instability should affect men and women alike. Evidence of sex differences in the association of sexual orientation with mixed lateral preferences, if corroborated, would thus render this factor overall unlikely.

### The Present Research

Directly addressing the shortcomings of previous research listed above, the present study investigated the associations of sexual orientation with lateral preferences among men and women, using two large and independent general population samples. Participants were not selected for sexual orientation or handedness, in order to avoid problems of selection bias and to get unbiased estimates of the association. In order to assess the replicability of our findings, we used a discovery and a replication sample. This approach is considered best practice in fields with hard-to-replicate evidence, such as genome-wide association studies (McCarthy et al., 2008), as it allows to safeguard against false-positive findings and to demonstrate the robustness of effects, if the replication attempt is successful.

We utilized validated multi-item inventories to assess handedness and footedness and used probabilistic methods (latent class analysis [LCA]; see Collins & Lanza, 2010) for the classification of lateral preferences. LCA has been used for the classification of lateral preferences by several independent groups of researchers (e.g., Büsch, Hagemann, & Bender, 2009; Dragovic & Hammond, 2007; Tran et al., 2014a; a recent overview of studies is provided by Sacco et al., 2018). Its main advantage lies in providing empirically derived classifications of lateral preference, instead of relying on ad hoc methods and arbitrary cutoffs. LCA uniquely also allows for checks of classification certainty (a concept similar to scale reliability for continuous measures). The use of multi-item inventories and LCA thus helped to maximize the reliability of assessment, and the validity of classification, of lateral preferences in this study and hence helped to maximize its statistical conclusion validity.

Drawing on the available evidence, we expected that the associations between sexual orientation and lateral preferences effectively obey the predictions of the GGT among women; i.e., bisexual/homosexual women show more non-right lateral preferences than heterosexual women, which may possibly reflect effects of higher prenatal testosterone levels. For men, we sought to test whether sexual orientation (bisexuality/homosexuality) shows any replicable association with lateral preferences at all. This investigation had a more explorative character, as the available evidence is contradicting and overall weaker. In carefully distinguishing between mixed and left preferences, we hoped to arrive at a clearer basis for deciding which of the competing theories (GGT, CH, or even developmental instability) might be best supported by the data.

In analysis, we controlled for sex-role identity and the number of older siblings. Mixed-handedness is associated with more feminine sex-role identity among men and more masculine sex-role identity among women (Tran, Stieger, & Voracek, 2014c; see also Lippa, 2003), but there is evidence that handedness is differentially associated with sexual attraction, behavior, and identity among men (Xu & Zheng, 2017). If associations with sexual orientation remain robust, controlling for sex-role identity, this would provide indirect evidence for separate pathways linking lateral preferences with the various components of sexual orientation. Further, the likelihood of homosexuality appears to increase with the number of older brothers (fraternal birth-order effect; e.g., Bogaert et al., 2018). Even though it is assumed that this effect holds for right-handed men only, some studies have reported associations for both men and women, with regard to both older brothers and sisters, and irrespective of handedness (e.g., Yule et al., 2014). Hence, the number of older brothers and sisters was also controlled for in the present study.

## Method

### Participants

This study used novel data from two large community samples, a discovery and a replication dataset. Table 1 presents descriptive characteristics of the two samples. The samples consisted of slightly more women than men with a broad age range. Regarding their educational background, around 5% (discovery sample) and 7% (replication sample) had completed lower secondary education, 71% and 75% upper secondary education, and 24% and 18% had completed some sort of tertiary education. Participants were chiefly of Austrian and German nationality. Overall, around 5% of participants reported non-heterosexual orientation. Across samples, more women than men identified themselves as bisexual (3.8% vs. 1.6%), whereas more men than women identified themselves as homosexual (2.9% vs. 1.4%). These numbers correspond closely to epidemiological data on the prevalence of bisexuality and homosexuality in the overall population (Chandra, Mosher, Copen, & Sionean, 2011).

**Table 1:** Sample descriptive statistics

	Sample 1 (discovery dataset)	Sample 2 (replication dataset)
*n*	2368	1565
Women, *n* (%)^a^	1257 (53%)	830 (53%)
Age, range (years)^b^	18–91	18–99
Interquartile range	23–41	23–46
Mean (*SD*)	32.86 (14.50)	34.21 (14.75)
Nationality, *n* (%)^c^		
Austria	1159 (49%)	846 (54%)
Germany	1063 (45%)	539 (35%)
Other	138 (6%)	168 (11%)
Sexual orientation^d^		
Heterosexual	2229 (95.0%)	1450 (94.8%)
Bisexual	58 (2.5%)	51 (3.3%)
Homosexual	47 (2.0%)	35 (2.3%)
Asexual	1 (< 0.1%)	3 (0.2%)
Other	12 (0.5%)	7 (0.5%)
Sex-role identity, mean (SD)^e^	− 0.17 (2.05)	− 0.20 (2.07)
Older brothers, range^f^	0–6	0–8
Mean (SD)	0.37 (0.65)	0.41 (0.71)
Older sisters, range^f^	0–5	0–5
Mean (SD)	0.37 (0.67)	0.39 (0.68)

Because of partially missing data, *n*s were ^a^2363 and 1562; ^b^2356 and 1560; ^c^2360 and 1553; ^d^2347 and 1546; ^e^2340 and 1543; ^f^2364 and 1565

### Measures

Handedness was assessed with a 10-item scale, comprising items of the Edinburgh Handedness Inventory (Oldfield, 1971) and of the Lateral Preferences Inventory (LPI; Coren, 1993). Footedness was assessed with an 8-item scale, comprising the 4 items of the LPI footedness scale and 4 items from Kalaycıoğlu, Kara, Atbaşoğlu, and Nalçacı (2008). Tran et al. (2014b) and Tran and Voracek (2018) provided evidence of the good psychometric properties and factorial validity of these composite scales; specifically, they also showed that the footedness scale comprises two dimensions: skilled footedness (5 items), i.e., unipedal tasks like kicking a ball; and movement footedness (3 items), i.e., bipedal tasks like stepping up stairs. Response options were *always right*, *usually right*, *no preference*, *usually left*, and *always left* (in this order; coded +2, +1, 0, -1, -2). Following psychometric evidence (Tran et al., 2014b), response options *always right* and *usually right*, as well as *usually left* and *always left* were each combined for further analysis and coded +1 and -1, respectively. Cronbach α in the two samples, with this coding applied, was 0.97/0.97 (handedness), 0.82/0.83 (skilled footedness), and 0.83/0.84 (movement footedness).

Sex-role identity was assessed with the Sex-Role Identity Scale (Storms, 1979), comprising three items each for M (masculinity) and F (femininity), rated on a 5-point scale. As in Tran et al. (2014c), we computed M–F (subtracting mean F from mean M scores) to obtain a bipolar measure of masculinity–femininity, ranging from +4 to -4. Cronbach α of M–F scores was 0.94/0.94 in the discovery/replication samples. Across the two samples, men (*M* = 1.46, *SD* = 1.35) and women (*M* = -1.63, *SD* = 1.38) differed by a large margin on M–F scores (Cohen *d* = 2.27; independent *t* test, *t* = 70.37, *df* = 3873, *p* < .001).

Sexual orientation was queried with one item in the sociodemographic section of the survey form. Response options were heterosexual, bisexual, homosexual, asexual, and other, as in Yule et al. (2014). For further analysis, bisexual and homosexual orientations were combined; individuals with asexual or other orientations were excluded (see Table 1). The sociodemographic section also contained questions on the numbers of both older and younger biological brothers and sisters (alive and deceased). We used these numbers to compute two birth-order indices which take family size into account that otherwise can distort observed associations (modified proportion of older brothers = (older brothers + 0.25)/(total siblings + 1), modified proportion of older sisters = (older sisters + 0.25)/(total siblings + 1); Blanchard, 2014). Right-handed bisexual/homosexual men did not differ significantly from right-handed heterosexual men regarding the number of older brothers in the two samples (*M* = 0.43, *SD* = 0.61 vs. *M* = 0.39, *SD* = 0.65; independent *t* test, *t* = 0.56, *df *= 1479, *p *= .574).

### Procedure

Data collection was crowdsourced and distributed among a total of 51 research assistants. This minimized recruitment bias and increased the sample heterogeneity vis-à-vis the underlying general population. For the two samples, data collection comprised two waves which were temporally separated by half a year and involved independent sets of data collectors. Participants were approached by the data collectors on a personal basis, using the data collectors’ personal contacts and word of mouth. Testing took place individually in quiet facilities and was carried out in majority with paper-and-pencil questionnaires, and electronically enhanced methods (i.e., PDF) otherwise. Participants had to be fluent in the survey language German; otherwise, there were no exclusion criteria.

### Analysis Plan

#### Classification of Lateral Preferences

Lateral preference ratings were analyzed with LCA. LCA employs a probabilistic model which searches for latent, discrete classes that explain the associations between observed variables (in the present case: answers to questionnaire items). We fitted models with 1, 2, 3, and 4 latent classes to the data, seeking the smallest number that explained the data best. For the evaluation of model fit, we utilized the Bayesian information criterion (BIC; lower values indicate better fit), the likelihood-ratio test of model fit (L^2^; preferably not significant for good fitting models), and percentages of classification error (lower values indicate better fit). LatentGOLD 4.5 (Vermunt & Magidson, 2005) was used for analysis.

#### Lateral Preferences and Sexual Orientation

Numbers of bisexual and homosexual individuals in the current study were, expectedly, only small and effects of lateral preferences were likely weak. Hence, we decided for an analysis plan that maximized statistical power, but still allowed to test for differential associations among men and women, as well as for the replicability of observed effects. Three regression models were tested in a two-stage approach of analysis. In Stage 1, lateral preferences were scored dichotomously, contrasting right with non-right preferences. Models of Stage 1 tested whether bisexuality/homosexuality was associated with lowered or elevated proportions of right preferences. Model 1.1 included only handedness, for comparison with prior related research findings. Model 1.2 also included the two dimensions of footedness, to test for their incremental validity and whether handedness remained significant, once measures of footedness were included. Model 1.3 included all lateral preferences that had remained significant in Model 1.2 and added the control variables of sex-role identity and birth order. All models included an interaction of lateral preferences with participant sex, and an interaction of the control variables participant sex and age. In Model 1.3, interactions of the control variables sex-role identity and birth order with sex were also included, as we expected sex differences, at least with regard to sex-role identity.

The models of Stage 2 served to test which lateral preferences (i.e., left or mixed; dummy-coded each) drive the associations observed in the Stage 1 models. We retested Models 1.1 and 1.3, to gain insight into the associations with respect to handedness (Model 2.1) and with respect to the lateral preferences in the final model (Model 2.3).

The replicability of lateral preference effects across Samples 1 (discovery data) and 2 (replication data) was tested via multi-group comparisons. All models were fitted to the data by constraining effect estimates to be equal across the two samples in a first step. In a second step, effects of lateral preferences that had been significant in the first step, plus their interactions with participant sex, were freely estimated in both samples. If this improved model fit significantly, separate effect estimates are reported for the two samples. Otherwise, joint effect estimates are reported and interpreted.

Conceptually, the statistical model was akin to a multi-group logistic regression analysis. For parameter estimation, the robust maximum likelihood method with Monte Carlo integration was used in Mplus 6.11 (Muthen & Muthen, 1998–2012). Replicability of laterality effects was tested with likelihood-ratio tests, contrasting the fit of the model with equality constraints versus the fit of the model with freely estimated parameters. Nominal significance was set to *p* < .05.

## Results

### Classification of Lateral Preferences

LCA models indicated for both samples three classes of handedness, of skilled footedness, and of movement footedness (Table 2). The mean posterior probabilities of assigning participants to these classes were high (handedness: 92% to 99%; skilled footedness: 88% to 95%; movement footedness: 93% to 96%), thus implying high classification certainty. Overall distributions of lateral preferences, separately for men and women, are presented in Table 3. Between the samples, lateral preferences did not differ among men and women (χ^2^ tests of independence, *p*s ≥ .475), except for skilled footedness among women (more right-footed, 72% vs. 67%, but fewer mixed-footed women, 19% vs. 26%, in Sample 1 than in Sample 2, χ^2^[2] = 11.65, *p* = .003) and for movement footedness among men (more mixed-footed, 30% vs. 25%, but fewer left-footed men, 16% vs. 20%, in Sample 1 than in Sample 2, χ^2^[2] = 7.76, *p* = .021). Within samples, men were replicably more often mixed-handed than women and replicably more often had mixed and left preferences in movement footedness than women (*z* tests, Bonferroni-corrected *p*s < .05). These differences are also clearly visible in Table 3.

**Table 2:** Fit of latent class models in the two samples

Number of latent classes	BIC	L^2^	*df*	*p*	% Classification error
*Handedness*					
1	19265.93	11325.32	2348	< .001	0.00
	13567.53	8110.71	1545	< .001	0.00
2	10745.21	2641.43	2327	< .001	0.14
	7791.15	2179.86	1524	< .001	0.34
3	9944.68	1677.74	2306	1.000	1.53
	7247.10	1481.34	1503	.650	1.71
4	9876.36	1446.25	2285	1.000	2.48
	7283.04	1362.81	1482	.987	2.20
*Skilled footedness*					
1	16464.58	3458.76	694	< .001	0.00
	11531.46	2644.56	718	< .001	0.00
2	14113.54	991.17	679	< .001	4.90
	9843.50	846.27	703	< .001	4.38
3	13556.48	348.65	668	1.000	8.05
	9417.64	339.49	692	1.000	8.78
4	13597.28	303.99	657	1.000	10.32
	9464.94	305.89	681	1.000	11.50
*Movement footedness*					
1	12908.07	2581.41	48	< .001	0.00
	8682.37	1942.85	44	< .001	0.00
2	11169.88	788.83	41	< .001	5.28
	7424.38	633.37	37	< .001	4.48
3	10482.03	46.59	34	.073	6.68
	6872.74	30.24	30	.453	5.14
4	10534.00	44.17	27	.020	16.20
	6909.72	15.73	23	.867	12.37

Entries in first lines apply to Sample 1 (discovery dataset), entries in second lines to Sample 2 (replication dataset). *BIC* Bayesian information criterion. *L*^2^ likelihood-ratio test of model fit. Following prior evidence (Tran & Voracek, 2018), the models for skilled footedness allowed for the correlated residuals of two items (‘trace a letter while standing’ and ‘erasing the letter’)

**Table 3:** Overall distributions of lateral preferences among men and women

	Men	Women
*Handedness*		
Right	1506 (82%)	1801 (86%)
Mixed	163 (9%)	122 (6%)
Left	169 (9%)	164 (8%)
*Skilled footedness*		
Right	1204 (66%)	1464 (70%)
Mixed	464 (25%)	454 (22%)
Left	170 (9%)	169 (8%)
*Movement footedness*		
Right	995 (54%)	1451 (70%)
Mixed	518 (28%)	416 (20%)
Left	325 (18%)	220 (11%)

Analysis *n* = 3925. Percent numbers rounded to the nearest integer and hence may not add up to 100%

### Non-Right Preferences and Sexual Orientation (Stage 1 Analysis)

**Fig. 1:**
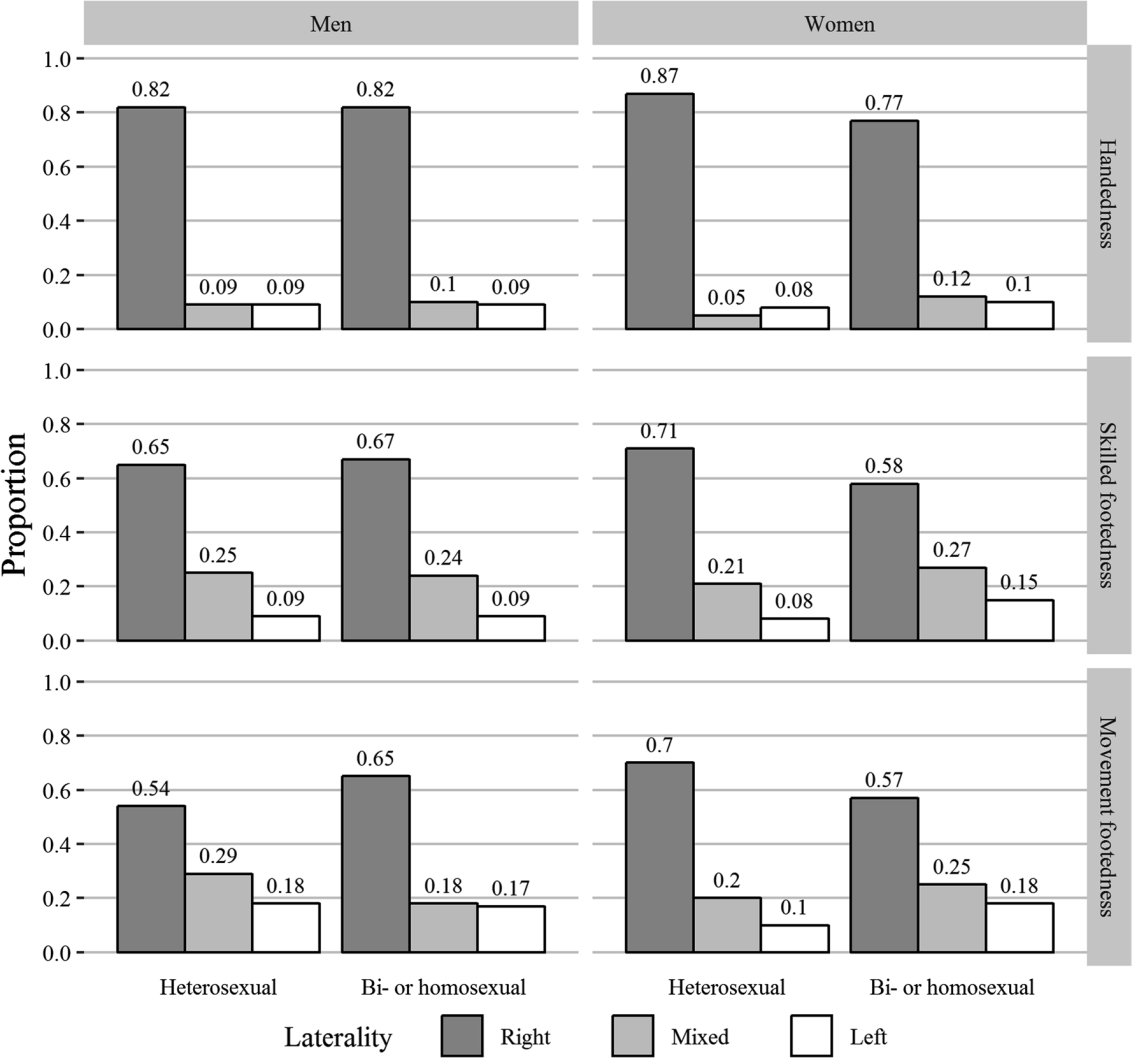
Distributions of lateral preferences among men and women, differentiated for sexual orientation

Descriptively, bisexual/homosexual women more often were non-right-handed and non-right-footed (skilled and movement footedness) than heterosexual women (Fig. 1). In contrast, bisexual/homosexual men more often had right preferences in movement footedness than heterosexual men. Among women, associations appeared to be driven by higher rates of mixed-handedness and, for footedness, by higher rates of mixed and left preferences. Among men, the association appeared to be driven by lower rates of mixed preferences.

Table 4 presents effect estimates (odds ratios) of the multi-group logistic regression models. Where interactions with participant sex were significant, main effect estimates in Table 4 applied only to women. Effect estimates for men were obtained by multiplying the main effect estimate with the corresponding interaction effect estimate and are provided in the main text. In all three models, younger participants were more likely to identify themselves as bisexual/homosexual than older participants (*p*s < 0.01). Further, effects of laterality were replicable across the two samples in all three models (not significant likelihood-ratio tests).

**Table 4:** Predictors of bisexuality/homosexuality in multi-group logistic regression analyses

	Model 1	Model 2	Model 3
Non-right-handed	**1.96 [1.22**–3.15]**	1.40 [0.82–2.39]	
Non-right-handed × participant sex	0.50 [0.23–1.06]	0.79 [0.34–1.84]	
Non-right-footed (skilled)		1.29 [0.82–2.01]	
Non-right-footed (skilled) × participant sex		0.78 [0.39–1.57]	
Non-right-footed (movement)		**1.55 [1.01**–2.41]*	**1.81 [1.21**–2.72]**
Non-right-footed (movement) × participant sex		**0.39 [0.20**–0.74]**	**0.38 [0.20**–0.71]**
*Control variables*			
Age	**0.98 [0.97**–0.99]***	**0.98 [0.97**–0.99]***	**0.98 [0.96**–0.99]**
Participant sex	0.99 [0.71–1.38]	1.45 [0.95–2.21]	1.07 [0.49–2.36]
Sex-role identity (M–F)			**1.60 [1.42**–1.80]***
Sex-role identity (M–F) × participant sex			**0.37 [0.31**–0.45]***
Older brothers			1.54 [0.57–4.13]
Older brothers × participant sex			1.05 [0.23–4.88]
Older sisters			0.63 [0.21–1.91]
Older sisters × participant sex			1.61 [0.29–8.94]
Likelihood-ratio test of replicability of laterality effects	Δ*χ*^2^ = 3.33, Δd*f* = 2, *p* = .189	Δ*χ*^2^ = 1.18, Δd*f* = 2, *p* = .556	Δ*χ*^2^ = 2.01, Δd*f* = 2, *p* = .366

Analysis *n* = 3870. Numbers are odds ratios with 95% confidence intervals. Right preferences served as reference category for effect tests of handedness and footedness. Significant effects (*p *< .05) are in boldface. *p < .05, **p < .01, ***p < .001

In Model 1.1, non-right-handedness was associated with sexual orientation (*p* = .006), but there was some indication that this effect might differ between men and women (interaction with sex: *p* = .071; see also Fig. 1). The test of this interaction effect was nominally not significant. However, it was likely underpowered, because of the highly skewed handedness distributions. Distributional differences between bisexual/homosexual and heterosexual individuals were readily apparent among women, but not among men (see Fig. 1). Descriptively, the effect estimate of non-right-handedness among men was *OR* = 0.97 [0.55–1.73], *p* = .927.

In Model 1.2, non-right-handedness lost its significance, but non-right preferences in movement footedness and their interaction with sex were significant (Table 4). The effect estimate of non-right preferences in movement footedness among men was *OR* = 0.61 [0.38–0.97], *p* = .036.

In Model 1.3, non-right preferences in movement footedness and their interaction with sex retained their significance in the presence of the control variables sex-role identity and number of older siblings. Bisexual/homosexual women reported a more masculine sex-role identity than heterosexual women, and bisexual/homosexual men reported a more feminine sex-role identity than heterosexual men (*OR* = 0.59 [0.51–0.69], *p* < .001). The number of older brothers and sisters appeared to be unrelated to sexual orientation in this model.

### Preferences Driving the Associations with Sexual Orientation (Stage 2 Analysis)

In Model 2.1, only mixed (*OR* = 2.56 [1.39–4.73], *p* = .003), but not left (*OR* = 1.50 [0.77–2.91], *p* = .234), preferences were linked with sexual orientation. The interaction of mixed-handedness with participants’ sex closely missed significance (*p* = .058). Descriptively, the effect estimate of mixed-handedness among men was *OR* = 0.99 [0.46–2.11], *p* = .973. The interaction of left-handedness with sex was not significant (*p* = .412). The effects of mixed-handedness and its interaction with sex generalized across the two samples (Δχ^2^ = 2.86, Δ*df* = 2, *p* = .240).

In Model 2.3, movement mixed-footedness was not significant (*OR* = 1.58 [0.98–2.55], *p* = .062), but its interaction with sex was (*p* = .008). Among men, the effect estimate of movement mixed-footedness was *OR* = 0.55 [0.30–1.01], *p* = .055. Movement left-footedness was significant (*OR* = 2.32 [1.33–4.04], *p* = .003), as was its interaction with sex (*p* = .027). Among men, the effect estimate of movement left-footedness was *OR* = 0.90 [0.49–1.66], *p* = .732. Sex-role identity and its interaction with sex retained their significance (*p*s < .001), whereas numbers of older siblings and their interactions with sex were not significant (*p*s ≥ .373). All effects of laterality and their interactions with sex generalized across the two samples (Δχ^2^ = 3.13, Δ*df* = 4, *p* = .536).

## Discussion

This study provided replicable evidence for an association of sexual orientation with handedness and footedness and showed that these associations of sexual orientation with lateral preferences differed between men and women. Bisexual/homosexual women were more likely mixed-handed and left-footed than heterosexual women and probably also more likely mixed-footed. In contrast, bisexual/homosexual men were more likely right-footed than heterosexual men. This association was most likely driven by lower rates of mixed preferences.

The findings add to the evidence that mixed, rather than left, preferences drive the associations of sexual orientation with handedness among women (Blanchard & Lippa, 2007; Ellis et al., 2017; Xu & Zheng, 2017). The lack of association of mixed and left preferences with sexual orientation among men is consistent with some previous evidence (e.g., Ellis et al., 2017; Yule et al., 2014), but contrary to some other findings (Blanchard & Lippa, 2007; Lalumière et al., 2000; Xu & Zheng, 2017). It is emphasized that the current study was based on a more reliable assessment and classification of lateral preferences than many prior studies. Regarding the observed increased likelihood for right preferences in footedness, the previously reported higher prevalence of extreme right-handedness among homosexual men (Bogaert, 2007; Kishida & Rahman, 2015) appears to provide converging evidence. Psychometric data (Tran et al., 2014b) suggest that the cutoffs used by studies to classify moderate and extreme right-handers may rather delineate an intermingled, combined group of mixed-handers and right-handers from ordinary right-handers.

Mixed-handedness is an indicator of developmental instability which has been previously considered to contribute to homosexuality (Lalumière et al., 2000). In the current study, mixed preferences were only more prevalent among bisexual/homosexual women, but not among men. We deem it thus unlikely that developmental instability was a relevant factor overall. Mixed- and left-footedness are also associated with testosterone-like effects on self-rated motor abilities (Tran & Voracek, 2016); they may thus point toward effects of high prenatal testosterone exposure as well. The current data thus appear to support the predictions of the GGT among women, but the predictions of the CH among men (assuming there is no association between callosal size and lateral preferences in bisexual/homosexual men). Further evidence for this conclusion is provided by the distributions of lateral preferences among men and women, and bisexual/homosexual and heterosexual individuals. Irrespective of sexual orientation, men reported more often mixed and left preferences than women in the current study. The distributions of lateral preferences themselves and this finding are in good agreement with previous data (e.g., Tran et al., 2014b; Tran & Voracek, 2018). This pattern was switched among the bisexual/homosexual men and women in the current study. Bisexual/homosexual women had a more male-typical distribution of lateral preferences, whereas bisexual/homosexual men a more female-typical distribution.

Thus, the current data appear to support the assumption of neurohormonal theory that high prenatal testosterone levels play a role in bisexuality/homosexuality among women, but low prenatal testosterone levels (or, alternatively, lower testosterone sensitivity) among men. Regarding the CH, we conclude that an association between corpus callosum size and handedness appears unlikely among homosexual men (as has been previously conjectured by Witelson et al., 2008). This prediction should be tested in more detail in further research, using reliable methods of handedness classification and also testing for associations with other lateral preferences, such as footedness.

We did not find indication of a birth-order effect on sexual orientation in our data. However, we included numbers of siblings only as a control variable into our models and did not aim to investigate patterns of association in a more detailed analysis. What can be concluded is that in our data birth order did not impact associations of lateral preferences with sexual orientation. This is consistent with other evidence from this research field, suggesting birth-order effects are solely applicable to right-handers (e.g., Blanchard & Lippa, 2007).

Sex-role identity and sexual orientation were associated in our data (more masculine sex-role identity among non-heterosexual women, more feminine sex-role identity among non-heterosexual men). However, this association had no noticeable effect on the associations of lateral preferences with sexual orientation. Thus, our data are consistent with other findings (e.g., Xu & Zheng, 2017) showing that associations of lateral preferences with sexual attraction, behavior, and identity are relatively independent of each other. Future research may benefit from assessing all three components of sexual orientation to gain a more detailed and complete picture of such associations.

### Possible Epigenetic Underpinnings

Mixed-handedness may have a heritability of up to 67% (Lien, Chen, Hsiao, & Tsuang, 2015), and familial data show that mixed-footed mothers more often have mixed- and left-footed children, indicating an X-linked mode of genetic transmission (Tran & Voracek, 2015). CAG repeat numbers in the androgen receptor (AR) gene on the X chromosome are indeed increased among mixed-handed men and non-right-handed women (e.g., Arming et al., 2015). Higher CAG repeat numbers are linked with decreased androgen receptor sensitivity for testosterone and thus are functional. This may be compensated among adult men by higher circulating testosterone levels (Huhtaniemi et al., 2009). However, among adult women, circulating testosterone levels are lower with higher CAG repeat numbers (Westberg et al., 2001).

There is evidence that sexually dimorphic development is canalized by sexually antagonistic epigenetic marks that increase testosterone sensitivity of the male fetus, but decrease testosterone sensitivity of the female fetus (Rice, Friberg, & Gavrilets, 2016). It has therefore been suggested that epigenetic marks from the opposite-sex parent escaping erasure during early development could be one potential cause of human homosexuality (Rice, Friberg, & Gavrilets, 2012).

Conceivably, a certain proportion of bisexual/homosexual women may have inherited higher CAG repeat numbers and, through paternal epigenetic marks, upregulated testosterone levels as well. This could explain the association of sexual orientation with non-right lateral preferences among women observed here and elsewhere and is also consistent with evidence for an association of CAG repeat numbers with non-right-handedness (Arming et al., 2015). Similarly, a certain proportion of bisexual/homosexual men may have inherited the antagonistic (maternal) epigenetic marks, in addition to, or independent of, a higher number of CAG repeats. As these men were less sensitive to the effects of testosterone during early development, no increase in non-right preferences occurred. The higher skewing of X chromosome inactivation (i.e., non-random inactivation of one of the two X chromosomes during early development) among mothers of homosexual than heterosexual sons (Bocklandt, Horvath, Vilain, & Hamer, 2006) could also play a role here.

It is emphasized that the current data are only indirect and the explanation proposed above only speculative. Also, our data do not rule out other pathways in the causation of bisexuality and homosexuality (such as the birth-order effect; see above). Specifically, they may explain sexual orientation only in a subgroup of men and women or in specific ethnicities (see Grimbos et al., 2010). However, our data are consistent with the epigenetic theory of homosexuality and suggest that it should be followed up. An empirical test of this theory has been formulated (Rice, Friberg, & Gavrilets, 2013), but results are still outstanding. A test of this theory could also provide evidence for whether low levels of prenatal testosterone, or rather lower testosterone sensitivity, is the mechanism driving this process in men.

### Limitations

Limitations of the current study pertain to the self-report nature of our data. Behavioral data may provide differing results from those obtained here. Assessment of sexual orientation relied on a single-item measure. Utilization of rating scales (e.g., the Kinsey Sexual Orientation Scale) or of multi-item scales, and assessing different components of sexual orientation, would have allowed for a more fine-grained analysis and for a cross-validation of sexual orientation ratings with sexual attraction. Albeit both our samples were large, the proportions of bisexual and homosexual individuals were, expectedly, only small, as were effects of lateral preferences. Thus, in analysis we could not differentiate bisexual from homosexual individuals. Bisexual and homosexual individuals may differ with regard to the distribution of lateral preferences (e.g., Xu & Zheng, 2017). Also, some effect tests in this study have been underpowered. Independent replications with even larger samples are still needed.

### Conclusions

This study provides evidence for sexually differentiated associations of lateral preferences with sexual orientation. Associations among women were consistent with predictions of the Geschwind–Galaburda theory, whereas those among men were consistent with predictions of the callosal hypothesis. Epigenetic mechanisms in conjunction with the functional CAG repeats-number polymorphism may account for the sexually differentiated patterns observed. We recommend utilizing reliable classification methods for lateral preferences in future research, to increase research efforts with regard to footedness, and empirical tests of the epigenetic theory of homosexuality.
